# Zoonotic diseases: understanding the risks and mitigating the threats

**DOI:** 10.1186/s12917-023-03736-8

**Published:** 2023-10-03

**Authors:** Ibrahim Elsohaby, Luca Villa

**Affiliations:** 1 Department of Infectious Diseases and Public Health, Jockey Club College of Veterinary Medicine and Life Sciences, City University of Hong Kong, Hong Kong SAR, China; 2 Centre for Applied One Health Research and Policy Advice (OHRP), City University of Hong Kong, Hong Kong SAR, China; 3 Department of Animal Medicine, Faculty of Veterinary Medicine, Zagazig University, Zagazig City, 44511 Sharkia Governorate Egypt; 4 Department of Veterinary Medicine and Animal Sciences, Università degli Studi di Milano, Via dell’Università, 6, Lodi, 26900 Italy

## Abstract

Zoonotic diseases are like a sneaky game of “tag” between animals and humans, where the stakes are high and the consequences can be deadly. From the bubonic plague to COVID-19, zoonotic diseases have affected humanity for centuries, reminding us of our interconnectedness with the animal kingdom and the importance of taking proactive measures to prevent their spread. Whether it is avoiding contact with animals or practicing good hygiene, staying safe from zoonotic diseases is a game we all need to play.

Zoonotic diseases, also known as zoonoses, are infectious diseases that can be transmitted from animals to humans. These diseases can be caused by bacteria, viruses, parasites, and fungi, and they can affect people of all ages and backgrounds. Zoonotic diseases can be transmitted to humans through direct contact with infected animals [1], through consumption of contaminated food or water [2], or through the bites of infected arthropod vectors such as ticks and mosquitoes [3]. In some cases, humans can also transmit zoonotic diseases to animals [4].

It is known that almost two-thirds of the pathogens that cause diseases in humans are of animal origin [5]. These diseases have a significant public health concern because they can cause severe illness and even death; besides, they also pose a significant economic burden on society due to the costs of medical treatment, lost productivity, and disease control measures.

Several risk factors associated with zoonotic disease infections have been described, but close contact with animals, including pets, livestock and wild animals is a crucial feature for zoonotic diseases transmission. Both direct contact with infected animals but also indirect contact in areas where animals live pose a risk for human health [1]. One of the classical examples that best illustrate the concept of direct zoonosis is rabies, which is transmitted by the bites of rabid carnivores to humans, particularly through exposure to domestic dogs. Sporothricosis is another example of an emerging disease that spreads by scratches of cats infected with the dimorphic fungus *Sporothrix brasiliensis*. Zoonotic scabies (pseudoscabies) is caused by burrowing mite *Sarcoptes scabiei*, which can infect humans through contact with affected animals. Indirect contact can also cause zoonotic diseases. *Bacillus anthracis*, a gram-positive bacterium with herbivorous animals as its reservoirs, can survive in the environment for decades and cause anthrax in humans. Additionally, some highly contagious zoophilic dermatophytes can infect humans directly, but they can also survive for extended periods in the environment and be transmitted indirectly through fomites [6].

Another chapter includes the foodborne and waterborne zoonotic diseases caused by the consumption of food or water contaminated by pathogenic microorganisms. With regard to food, the risk of contamination occurs at any point along the chain “from farm to fork”. The most common foodborne diseases are caused by *Campylobacter*, *Salmonella*, *Yersinia*, Shiga toxin-producing *Escherichia coli* (STEC) and *Listeria monocytogenes* [7]. Among bacterial zoonoses, the consumption of unpasteurized milk is implicated in the human transmission of *Mycobacterium bovis* and *Brucella* spp., which are the etiological agents of tuberculosis and brucellosis, respectively. Recently, pigs and pork products were implicated in zoonotic transmission of Hepatitis E Virus to humans, and the incrimination of also other foods was suspected. Food-borne parasites include nematodes (*Anisakis* spp. in marine fish and *Trichinella* spp. in pigs, horses, and wild mammals), cestodes (*Taenia saginata* in cattle and *Taenia solium* in pigs), and trematodes (*Opisthorchis* spp. in fish and *Paragonimus* spp. in crustaceans), but also protozoa, in particular *Toxoplasma gondii*, infecting one-third of the human population worldwide. Concerning water, cryptosporidiosis and giardiasis are acute gastrointestinal infections caused by the protozoa *Cryptosporidium* and *Giardia* species, spread to humans via drinking, food processing, and recreational use of contaminated water [8].

In recent years, climate change has influenced zoonotic diseases through alterations in host, vector, and pathogen dynamics. Global warming and geoclimatic variations are associated with ecological shifts affecting the current global incidence and distribution of vector-borne diseases transmitted by the bite of infected arthropods. Mosquitos are traditionally implicated in the transmission of *Plasmodium* parasites, the causative agent of malaria in humans, but can also transmit other emerging viral diseases, including West Nile, Chikungunya, Zika and Dengue. Recently, sandflies rapidly established in Northern regions, leading to the change in distribution of leishmaniasis, a protozoan infection caused by *Leishmania infantum*, in both animal and human hosts. Besides, increased temperatures impact both ticks and host species populations: among tick-borne zoonoses, *Ixodes* species acts as both reservoir and vector for Lyme Borreliosis, caused by *Borrelia burgdorferi* spirochetes, and also tick-borne encephalitis (TBE) viruses. Examples of other vectors responsible for transmitting zoonotic diseases include fleas (Plague), mites (Scrub typhus), triatomine bugs (Chagas disease), black (Onchocerciasis) and tsetse (Trypanosomiasis) flies [9].

Emerging zoonoses are defined as zoonoses that are newly recognised or newly evolved, or that have occurred previously but show an increase in incidence or expansion in geographical, host or vector range, socio-economic, whose origins are correlated with socio-economic, environmental and ecological factors. Among these, it is noteworthy to mention the role of wild animals in the transmission, amplification and zoonotic overflow and dissemination of etiological agents, as in the case of the Coronaviruses causing Middle East Respiratory Syndrome (MERS), the Severe Acute Respiratory Syndrome (SARS) and the most recent Coronavirus pandemic (COVID-19) [10].

Certain populations may be at higher risk for zoonotic diseases, such as people who work with animals or in rural areas, children, the elderly, and individuals with weakened immune systems; the disproportionate impact on vulnerable communities, especially those living in poverty or with limited access to healthcare, should be considered. Moreover, global changes, such as climate change, habitat destruction, deforestation, changes in land use, intensification of agriculture, and increased travel and trade, can disturb the delicate balance between humans, animals, and the environment, leading to an increased risk of zoonotic disease transmission to communities such as farmers and rural dwellers, wildlife workers and conservationists, and indigenous populations [11].

In recent years, advances in the field of zoonotic disease research, with regard to early detection diagnosis, appropriate treatment, prophylaxis and control, were achieved. For instance, the use of polymerase chain reaction (PCR) technology has revolutionized the detection of zoonotic viruses such as Ebola and Zika, enabling healthcare professionals to diagnose these diseases rapidly. Additionally, significant strides have been made in the development of vaccines and treatments for zoonotic diseases, such as the development of an effective vaccine for Ebola and Nipah virus. Similarly, the development of new treatments for diseases like West Nile virus and avian influenza and the recent discovery of a new antiviral drug that could be effective against multiple zoonotic viruses, including Ebola, Lassa fever, and COVID-19 could helped to save numerous lives. Furthermore, the advancements in CRISPR and machine learning algorithms technology have significantly contributed to advancing our understanding of zoonotic diseases, also to predict the emergence of new zoonotic diseases, potentially allowing for earlier detection and prevention.

However, there is still a need to improve global surveillance and reporting systems to enable prompt identification and response to outbreaks: indeed, these gaps in research underscore the need for continued investment in zoonotic disease research to safeguard public health and prevent future pandemics. Preventing zoonotic diseases requires a coordinated effort between public health officials, veterinarians, and other professionals. This includes measures such as surveillance and early detection, vaccination programs for animals, proper food handling and preparation, and education and awareness campaigns for the general public. The One Health approach promotes collaboration among professionals from different fields, including human medicine, veterinary medicine, environmental science, and public health. It involves sharing information and resources and developing integrated surveillance systems to detect and monitor zoonotic diseases in both humans and animals [12]. The commitment emphasizes the importance of a One Health approach to prevent and control the transmission of zoonotic diseases. It also promotes joint action between the three organizations to improve zoonotic disease surveillance and support research to better understand the drivers of zoonotic disease emergence and spread.

Overall, we aim to highlight the importance of ongoing investment in zoonotic disease research by launching this collection and inspire continued efforts to address this pressing public health challenge. By highlighting recent advances, we can raise awareness about the progress that has been made and the work that still needs to be done, and hopefully motivate further investment in this critical area of research.

## Data Availability

Not applicable.

## References

[1] Rahman M, Sobur M, Islam M, Ievy S, Hossain M, El Zowalaty ME, Rahman A, Ashour HM (2020). Zoonotic diseases: etiology, impact, and control. Microorganisms.

[2] Loh EH, Zambrana-Torrelio C, Olival KJ, Bogich TL, Johnson CK, Mazet JA, Karesh W, Daszak P (2015). Targeting transmission pathways for emerging zoonotic disease surveillance and control. Vector-Borne and Zoonotic Diseases.

[3] Kilpatrick AM, Randolph SE (2012). Drivers, dynamics, and control of emerging vector-borne zoonotic diseases. The Lancet.

[4] Messenger AM, Barnes AN, Gray GC (2014). Reverse zoonotic disease transmission (zooanthroponosis): a systematic review of seldom-documented human biological threats to animals. PLoS ONE.

[5] Abebe E, Gugsa G, Ahmed M. Review on major food-borne zoonotic bacterial pathogens. *Journal of tropical medicine* 2020, 2020. 10.1155/2020/4674235 PMC7341400 32684938

[6] Carpouron JE, de Hoog S, Gentekaki E, Hyde KD (2022). Emerging Animal-Associated Fungal Diseases. J Fungi.

[7] Authority EFS (2022). Prevention ECfD, Control: the European Union One Health 2021 Zoonoses Report. EFSA J.

[8] Rupasinghe R, Chomel BB, Martínez-López B (2022). Climate change and zoonoses: a review of the current status, knowledge gaps, and future trends. Acta Trop.

[9] Chomel BB (2008). Control and prevention of emerging parasitic zoonoses. Int J Parasitol.

[10] Cupertino MC, Resende MB, Mayer NA, Carvalho LM, Siqueira-Batista R (2020). Emerging and re-emerging human infectious diseases: a systematic review of the role of wild animals with a focus on public health impact. Asian Pac J Trop Med.

[11] Allen T, Murray KA, Zambrana-Torrelio C, Morse SS, Rondinini C, Di Marco M, Breit N, Olival KJ, Daszak P (2017). Global hotspots and correlates of emerging zoonotic diseases. Nat Commun.

[12] Ghai RR, Wallace RM, Kile JC, Shoemaker TR, Vieira AR, Negron ME, Shadomy SV, Sinclair JR, Goryoka GW, Salyer SJ (2022). A generalizable one health framework for the control of zoonotic diseases. Sci Rep.

